# HEALTH-RELATED QUALITY OF LIFE IN CHRONIC LIVER DISEASE: CLINICAL BURDEN, DETERMINANTS, AND IMPLICATIONS FOR PATIENT-CENTERED CARE

**DOI:** 10.1590/S0004-2803.24612026-064

**Published:** 2026-08-17

**Authors:** Maria Gabriela Fernandes DEZAN, Caio Pedro Gomes da HORA, Catharina Ribeiro LYRA, Lourianne Nascimento CAVALCANTE, Helma Pinchemel COTRIM, André Castro LYRA

**Affiliations:** 1 Universidade Federal da Bahia, Hospital Universitário Professor Edgard Santos (HUPES), Serviço de Gastro-Hepatologia, Salvador, BA, Brasil.; 2 Universidade Federal da Bahia, Programa de Pós-Graduação em Medicina e Saúde, Salvador, BA, Brasil.; 3 Instituto Do’r de Ensino e Pesquisa / Hospital São Rafael, Serviço de Gastro-Hepatologia, Salvador, BA, Brasil.; 4 Escola Bahiana de Medicina e Saúde Pública, Salvador, BA, Brasil.

**Keywords:** Health-related quality of life, cirrhosis, chronic liver disease, quality of life tools, Qualidade de vida relacionada à saúde, cirrose, doença hepática crônica, instrumentos de avaliação da qualidade de vida

## Abstract

**Background:**

Chronic liver disease significantly distresses patients’ health-related quality of life (HRQoL). Factors such as ascites, hepatic encephalopathy, medication side effects, malnutrition, sarcopenia, frailty, the prospect of liver transplantation, caregiver reliance, financial burdens, and future uncertainty can all negatively influence HRQoL. Evaluating HRQoL in liver disease is critical. Recognizing factors that impair HRQoL in these patients enables the multidisciplinary team to promote health in a comprehensive biopsychosocial approach to well-being.

**Methods:**

We searched the PubMed database using these descriptors: health-related quality of life, cirrhosis, liver disease, alcohol-related liver disease, metabolic dysfunction-associated steatotic liver disease, viral hepatitis, autoimmune hepatitis, primary biliary cholangitis, primary sclerosing cholangitis, hepatocellular carcinoma, liver transplantation, sarcopenia, frailty, and malnutrition.

**Results:**

HRQoL is negatively affected by the severity of liver disease and its complications. Moreover, nutritional and psychological aspects such as sarcopenia, frailty, malnutrition, fear of disease progression and need for transplantation, feeling of dependence on caregivers, and financial impacts of the disease also culminate in reduced quality of life.

**Conclusion:**

People with chronic liver disease experience a significant impairment in their quality of life due to various clinical, nutritional, and social factors. For this reason, a multidisciplinary, global assessment with systematic appreciation of HRQoL at each consultation is essential when following these patients.

## HIGHLIGHTS

• Tools that measure HRQoL in liver patients include CLDQ, LDSI 2.0, and LDQOL 1.

• Disease severity, ascites, and encephalopathy are associated with worse HRQoL.

• HRQoL assessment may help predict hospitalization in chronic liver disease.

• Routine HRQoL assessment supports biopsychosocial care in CLD subjects.

## RESUMO

**Contexto:**

A doença hepática crônica afeta significativamente a qualidade de vida relacionada à saúde (QVRS) dos pacientes. Fatores como ascite, encefalopatia hepática, efeitos colaterais de medicamentos, desnutrição, sarcopenia, fragilidade, perspectiva de transplante hepático, dependência de cuidadores, encargos financeiros e incerteza quanto ao futuro podem influenciar negativamente a QVRS. Avaliar a QVRS em doenças hepáticas é fundamental. Reconhecer os fatores que comprometem a QVRS nesses pacientes permite que a equipe multidisciplinar promova a saúde por meio de uma abordagem biopsicossocial abrangente para o bem-estar.

**Métodos:**

Realizamos uma busca na base de dados PubMed utilizando os seguintes descritores: qualidade de vida relacionada à saúde, cirrose, doença hepática, doença hepática relacionada ao álcool, doença hepática esteatótica associada à disfunção metabólica, hepatite viral, hepatite autoimune, colangite biliar primária, colangite esclerosante primária, carcinoma hepatocelular, transplante hepático, sarcopenia, fragilidade e desnutrição.

**Resultados:**

A QVRS é afetada negativamente pela gravidade da doença hepática e suas complicações. Além disso, aspectos nutricionais e psicológicos como sarcopenia, fragilidade, desnutrição, medo da progressão da doença e da necessidade de transplante, sentimento de dependência de cuidadores e impactos financeiros da doença também culminam em redução da qualidade de vida.

**Conclusão:**

Pessoas com doença hepática crônica apresentam comprometimento significativo na qualidade de vida devido a diversos fatores clínicos, nutricionais e sociais. Por essa razão, uma avaliação multidisciplinar e abrangente, com avaliação sistemática da qualidade de vida relacionada à saúde em cada consulta, é essencial no acompanhamento desses pacientes.

## INTRODUCTION

**FIGURE 1:**
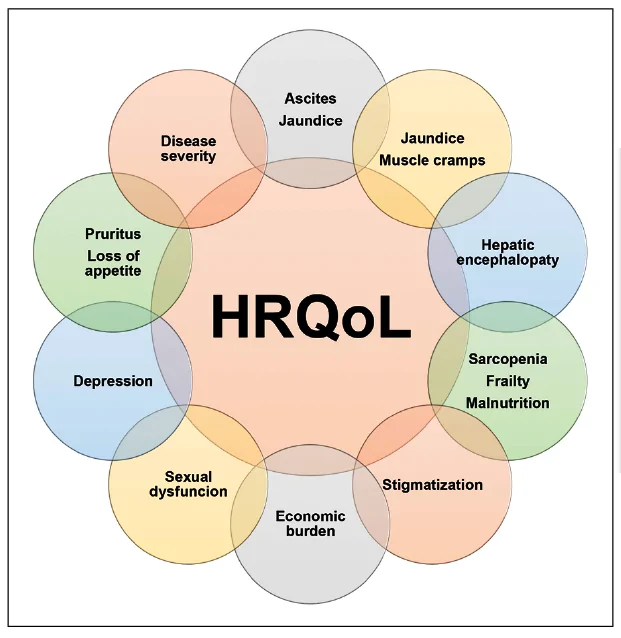
Factors associated with impaired HRQoL in chronic liver disease.

In 1947, the World Health Organization (WHO) broadened the definition of health. Health became more than just the absence of illness; it encompassed an individual’s overall well-being and was defined as a complete state of physical, mental, and social well-being^1^. Quality of life results from both positive and negative life experiences. From a health perspective, it reflects how symptoms, complications, and stigma of disease affect a person’s functionality, economic situation, social support, and mental well-being. Within this context, chronic liver disease (CLD) is prevalent worldwide due to diverse causes. These include diseases inherent to individuals, such as hemochromatosis (HH), Wilson’s disease (WD), autoimmune hepatitis (AIH), primary biliary cholangitis (PBC), and primary sclerosing cholangitis (PSC). Acquired health issues, such as alcohol-related liver disease (ARLD), viral hepatitis B (HBV), hepatitis C (HCV), and metabolic dysfunction-associated steatotic liver disease (MASLD), also contribute. CLD typically has a long natural history. It is characterized by numerous complications, symptoms, and signs that can significantly affect patients’ health-related quality of life (HRQoL). These factors also directly influence their families and the overall health system^2^ ^,^ ^3^. Patients with chronic liver disease may suffer from specific complications that ultimately impair quality of life. Examples include hepatic encephalopathy, ascites, and variceal hemorrhage. They may also experience other symptoms, such as pruritus, pain, depression, loss of appetite, sexual dysfunction, and loss of social interaction. The disease can impair overall well-being and life satisfaction^4^. Figure 1 summarizes the main factors associated with health-related quality of life (HRQoL) in patients with chronic liver disease. Therefore, understanding the impact of liver disease and its complications on health-related quality of life becomes essential. Despite its significance, physicians often neglect this issue during patient assessment.

HRQoL: health-related quality of life.

## METHODS

This narrative review comprehensively evaluated the scientific literature on the primary tools used to evaluate health-related quality of life and its association with clinical, nutritional, psychological, and financial parameters. Between March 2025 and November 2025, we searched the PubMed database using relevant descriptors and Boolean operators.

### HRQoL Measurement Tools

Measuring HRQoL is challenging because it requires analyzing subjective perceptions alongside objective disease-related parameters^3^. Effective instruments must detect relevant changes over time, be reliable, be free of random errors, and be validated^1^. Generic scales such as the Short Form-36 (SF-36), Nottingham Health Profile (NHP), and Sickness Impact Profile (SIP) allow the assessment of the relative impact that a given health condition can have on the individual, thus functioning as a guide for public health policies^3^ ^,^ ^4^. The SF-36 consists of 36 questions grouped into eight domains (functional capacity, physical aspects, pain, general health status, vitality, social and emotional aspects, and mental health). The final score ranges from 0 to 100, and the higher the score, the higher the quality of life^5^ ^,^ ^6^. The NHP is a 38-question questionnaire divided into six groups: pain, physical mobility, emotional reactions, energy level, social isolation, and sleep. Each question has a “yes” or “no” response, and each category receives a final score ranging from 0 to 100. A low NHP score corresponds to a better quality of life^7^. The SIP is a questionnaire, scored by percentage (0-100%), that assesses an individual’s perception of their health, consisting of 136 items encompassing three dimensions: physical (ambulation, mobility, and self-care), psychosocial (social interaction, alert behaviors, emotional behavior, and communication) and independent categories (sleep and rest, food, work, home maintenance, recreation). The higher the percentage achieved, the greater the individual’s dysfunction^8^. There is also the EuroQoL-5 dimension (EQ-5D), which assesses five dimensions (mobility, self-care, usual activities, pain/discomfort, and anxiety/depression), with the following gradations for each dimension: absence, slight, moderate, severe, and extreme^9^. However, in chronic liver disease (CLD), disease-specific HRQoL instruments are necessary to more accurately capture the impact of symptoms and clinical features unique to this group of conditions over time. Questionnaires such as the Chronic Liver Disease Questionnaire (CLDQ) and the Liver Disease Quality of Life 1.0 (LDQOL 1.0) are widely used to assess quality of life in this population. The CLDQ, the most commonly used liver-specific instrument, can be applied across different etiologies and stages of liver disease. It consists of 29 items grouped into six domains: abdominal symptoms, fatigue, systemic symptoms, activity, emotional function, and worry. Each item is scored on a 1-7 scale according to the frequency of symptoms or feelings, with higher scores indicating better quality of life^10^ ^,^ ^11^. The LDQOL 1.0 includes a generic core based on the SF-36, along with 75 liver disease-specific items, and is most often used in patients with advanced cirrhosis^12^. Another disease-specific instrument is the Liver Disease Symptom Index 2.0 (LDSI 2.0), which comprises 18 items covering nine domains; however, it has been validated only in the Netherlands^3^ ^,^ ^4^. The main characteristics of these instruments are summarized in Tables 1 and 2.

**TABLE 1:** Characteristics of the main generic HRQoL scales^3^ ^-^ ^9^.

	NHP	SIP	EQ-5D
Nº of items	38	136	5
Nº of domains/subescales	7	12	5
Domains/subescales	- Energy;	- Ambulation;	- Mobility;
	- Pain;	- Body care/movement;	- Self-care;
	- Emotional reactions;	- Mobility;	- Usual activity;
	- Sleep;	- Social interaction;	- Pain/discomfort;
	- Social isolation;	- Alertness behavior;	- Anxiety/depression.
	- Physical mobility.	- Emotional behavior;	
		- Communication;	
		- Sleep and rest;	
		- Eating;	
		- Work;	
		- Home management;	
		- Recreation/pastimes.	
Comments	- Generic scale less frequently used for liver disease;	- No validation for other languages;	- Quick and simple answers;
	- Validation in other languages;	- Widely used; It can be tiring;	- Self-administered;
	- Quick responses;	- Higher scores mean better HRQoL.	- There is no need for a face-to-face interviewer.
	- Higher scores represent greater impairment of HRQoL.		

EQ-5D: EuroQol-5D. HRQoL: health-related quality of life. NHP: Nottingham health profile. SF-36: short-form 36. SIP: sickness impact profile

**TABLE 2:** Characteristics of the main liver-specific HRQoL scales^3^ ^,^ ^4^ ^,^ ^10^ ^-^ ^12^.

	CLDQ	LDQOL	LDSI 2.0
Nº of items	29	101	18
Nº of domains/subescales	6	20 (12 of its own + SF-36)	9
Domains/subescales	- Fatigue;	- CLD-related symptoms;	- Itch;
	- Activity;	- CLD-related effects on activities of daily living;	- Joint pain;
	- Emotional function;		- Pain in the right upper abdomen;
	- Abdominal symptoms;	- Concentration;	- Sleepiness during day;
	- Systemic symptoms;	- Memory;	- Worry about family situation;
	- Worry.	- Sexual functioning;	- Decreased appetite;
		- Sexual problems;	- Depression;
		- Sleep;	- Fear of complications;
		- Loneliness;	- Jaundice.
		- Hopelessness;	
		- Qual. of social interaction;	
		- Health distress;	
		- Self-perceived stigma of CLD.	
Comments	- Validated use in several countries;	- More sensitive than CLDQ;	- Only validated in the Netherlands;
	- Widely used;	- It can be tiring;	- Not widely used.
	- More widely known;	- Data was published only in English.	
	- Simple to perform;		
	- Loses sensitivity in assessing discrete variations in HRQoL.		

CLD: chronic liver disease. CLDQ: chronic liver disease questionnaire. HRQoL: health-related quality of life. LDQOL: Liver Disease Quality of Life. LDSI: Liver Disease Symptom Index.

### Predictors of HRQoL in chronic liver disease: sociodemographic factors, disease severity, and symptoms

Health-related quality of life (HRQoL) is markedly reduced in individuals with chronic liver disease compared to the general population, with patients with cirrhosis showing the lowest scores^3^. This decline reflects not only disease progression but also sociocultural factors and the stigma associated with liver disease. Multiple elements contribute to impaired HRQoL in cirrhotic patients, including limited social support, depression, sleep disturbances, frailty, cramps, memory loss, fatigue, falls, malnutrition, cognitive impairment, hepatic encephalopathy, muscle cramps, and disease severity as measured by Child-Pugh and MELD scores. A comprehensive understanding of these determinants is essential to guide both preventive and therapeutic interventions^3^ ^,^ ^13^ ^,^ ^14^ ^,^ ^15^.

Janani et al. evaluated 149 patients using the SF-36 and CLDQ questionnaires and found that those aged ≤45 years reported lower HRQoL across all CLDQ domains. The predominance of Child C patients with higher MELD values in this age group - along with ascites and hepatic encephalopathy, which also negatively affect HRQoL - likely explains these findings^16^. Les et al., in a study of 212 cirrhotic patients, reported that female sex, non-alcoholic etiology, ascites, and hypoalbuminemia independently impaired HRQoL on the CLDQ, while previous hepatic encephalopathy, ascites, and low hemoglobin were the main predictors on the SF-36^17^. Other studies show that ascites, abnormal upper endoscopy findings (esophageal varices and/or portal hypertensive gastropathy), active smoking, female sex, hypoalbuminemia, younger age, prolonged prothrombin time, hepatic encephalopathy, and higher Child-Pugh and MELD scores correlated with worse HRQoL^18^ ^,^ ^19^ ^,^ ^20^.

Arguedas et al. evaluated 160 patients using the SF-36 questionnaire and reported that hepatic encephalopathy adversely affected HRQoL regardless of Child-Pugh score, with the greatest impact on emotional limitations, mental health, and social functioning^21^. Conversely, Sanyal et al. demonstrated that rifaximin improved HRQoL in patients with cirrhosis and recurrent hepatic encephalopathy^22^. Additional studies showed that rifaximin also enhanced quality of life and cognitive function in individuals with minimal hepatic encephalopathy^23^. Lactulose has likewise been associated with improvements in HRQoL among patients with cirrhosis^24^.

Another symptom closely linked to impaired quality of life is pruritus. A systematic review examining pruritus in patients with cholestatic diseases reported a significant association between this symptom and HRQoL, particularly affecting the parameters of physical and emotional limitations, bodily pain, vitality, energy, and physical mobility^25^. Fatigue has been described in liver diseases such as primary biliary cholangitis (PBC), hepatitis C, and metabolic dysfunction-associated steatotic liver disease (MASLD). Fatigue significantly reduces quality of life, and importantly, treating the underlying liver condition does not always lead to its complete resolution^26^. Ascites significantly impairs the quality of life in patients with liver cirrhosis. A Spanish study involving 523 individuals with cirrhosis and ascites assessed HRQoL using the SF-36 and identified several physical factors associated with worse outcomes, including non-alcoholic etiology, severe ascites, previous hepatic encephalopathy, falls, lower limb edema, and hyponatremia. Regarding the mental component, only hyponatremia and the use of lactulose were linked to reduced quality of life^27^.

Some therapeutic measures and their impact on the quality of life are under investigation. In a clinical trial, Stepanova et al. randomized 60 patients with refractory ascites to either large-volume paracentesis or an alfapump®-an implantable, programmable device that transfers ascitic fluid from the abdominal cavity to the bladder-and compared HRQoL using the SF-36 and CLDQ. The alfapump® was independently associated with improved quality of life at 3 months^28^. Similarly, another study of 30 patients with cirrhosis and ascites found that the alfapump® reduced the need for repeated large-volume paracentesis and enhanced HRQoL as measured by the CLDQ^29^.

### HRQoL and the etiology of liver disease

Although existing literature indicates that the etiology of liver disease may influence health-related quality of life (HRQoL), the evidence does not support a consistent or well-defined pattern of association. This inconsistency may arise from the confounding effects of external factors and methodological differences among studies^3^. Therefore, it is crucial to evaluate the impact of each specific etiology of liver disease to better understand its effects on HRQoL.

### Alcohol-related liver disease

Elde and colleagues evaluated the relationship between comorbidities, alcohol use, smoking, and quality of life (assessed by the SF-12 questionnaire) in individuals with alcohol-related liver disease. Among the 772 individuals included, the authors observed that chronic obstructive pulmonary disease, musculoskeletal diseases, smoking, and active alcoholism were associated with worse quality of life, regardless of the severity of liver cirrhosis^30^. Another study evaluated the impact of alcohol use on the quality of life of patients with liver cirrhosis (N=329) using the LDQoL tool and found that problematic drinking (defined by ≥8 on the Alcohol Use Disorders Identification Test) was associated with the worse overall quality of life, especially in the memory/concentration and health distress subscales, regardless of the severity of liver disease^31^. Singh et al. demonstrated that quality of life decreases as cirrhosis becomes more severe, especially in those individuals with alcohol dependence and malnutrition^32^.

### Metabolic dysfunction-associated steatotic liver disease

Individuals living with metabolic dysfunction-associated steatotic liver disease (MASLD) may have their quality of life impaired by several factors. Many of them are obese and have other metabolic diseases that impair their general physical health and mobility. Obesity leads to the stigmatization of these individuals in society, which contributes to deleterious psychosocial effects on the perception of quality of life. The recent change in nomenclature from non-alcoholic fatty liver disease (NAFLD) to MASLD did not appear to influence data related to quality of life. Hashida et al. found in their study that 99.3% of patients with NAFLD met the criteria for MASLD, and the CLDQ-NAFLD/NASH questionnaire yielded similar scores between the two groups^33^. A multicenter study with 2,117 patients with MASLD from 24 countries applied the CLDQ-NASH questionnaire and another questionnaire to assess liver disease burden and found that 9% of respondents related the stigma of the disease to the diagnosis of MASLD and 24% to the presence of obesity, with the experience of stigmatization or discrimination being an independent factor for worse HRQoL in these individuals^34^. Another multicenter, prospective study of 513 patients with metabolic steatosis disease who underwent the EQ-5D and CLDQ questionnaires found that people with the disease had a worse quality of life than the general population; female gender, type 2 diabetes mellitus, and depression were associated with worse EQ-5D scores. There was no difference in HRQoL between patients with and without advanced fibrosis^35^. Finally, in a systematic review, Assimakopoulos and colleagues evaluated 14 studies involving almost 5,000 patients; the authors concluded that metabolic dysfunction-associated steatotic liver disease does impair HRQoL, mainly when associated with factors such as female gender, advanced age, presence of fatigue, cirrhosis, obesity, and diabetes mellitus^36^. Some treatment-related factors may also influence the quality of life of patients with MASLD. A multicenter, randomized, double-blind, placebo-controlled British investigation evaluated the efficacy and safety of liraglutide in patients with steatohepatitis, with quality of life as a secondary outcome. The authors found that liraglutide significantly improved quality of life, as measured by the SF-36^37^. Sanyal and colleagues randomized 247 patients with steatohepatitis without diabetes to three groups: pioglitazone, vitamin E, or placebo, and, as a secondary outcome, evaluated HRQoL (using the SF-36). They found no differences across any domain among the three groups^38^. Conversely, semaglutide has been shown to improve quality of life, as reported by Romero-Gómez et al. in a randomized trial of 320 individuals with biopsy-proven steatohepatitis. In this study, the use of semaglutide was associated with significant improvements in the physical components assessed by the SF-36^39^. Recently approved by the Food and Drug Administration (FDA), resmetirom has also shown positive effects on quality of life. Younossi et al. found improvements in physical function, vitality, and mental health in individuals with steatohepatitis treated with resmetirom^40^. On the other hand, weight loss is a non-pharmacological approach that also appears to improve the quality of life of MASLD patients. An American study found that losing at least 5% of body weight significantly improved the CLDQ quality-of-life score, with better results in the domains of fatigue, abdominal symptoms, activity, and worry. A 5-point reduction in body mass index (BMI) was associated with an adjusted 10% improvement in quality of life^41^.

### Chronic viral hepatitis B and C

Chronic hepatitis C significantly impairs HRQoL even without associated liver fibrosis. Factors such as advanced age, female gender, and depressive symptoms appear to be associated with worse quality of life among HCV subjects^42^. Björnsson et al. used the SF-36 to assess individuals with hepatitis C. They evaluated HCV patients without cirrhosis, with compensated and decompensated cirrhosis, a group of compensated and decompensated cirrhosis due to other etiologies, and one group of HCV individuals with sustained virological response (SVR) without cirrhosis. Patients with hepatitis C had impaired HRQoL, and its worsening accompanied the severity of the disease^43^. SVR is associated with a reduction in HCV-related complications and mortality and appears to improve the quality of life of these individuals, according to some studies^44^ ^,^ ^45^. Conversely, Miyasaka et al. did not detect any difference in the perception of quality of life (using the SF-8) after SVR when evaluating 109 individuals with hepatitis C treated with regimens containing sofosbuvir^46^. Quality of life data related to interferon treatment showed that although there was a decrease in quality of life during treatment (mainly in parameters such as physical, emotional, social, and vitality), 12 to 24 weeks after the end of therapy, patients showed an improvement in HRQoL compared to the pre-treatment period^47^. Interestingly, this improvement in HRQoL does not occur immediately after treatment, as Pojoga et al. reported no improvement in quality of life measured immediately after treatment completion^48^.

Hepatitis B virus remains a significant public health concern that adversely affects the quality of life of individuals, particularly in advanced disease stages characterized by impaired liver function or cancer development. The severity of liver disease, presence of hepatocellular carcinoma (HCC), and active disease phases are associated with reduced quality of life^49^ ^,^ ^50^. Xue et al. investigated the impact of antiviral therapy on quality of life, finding that antiviral treatment improves health-related quality of life (HRQoL)^51^, primarily in the physical domain, with minimal effect on psychological parameters. Additional factors contributing to diminished quality of life among hepatitis B patients include depression, anxiety, disease-related stigma, and fatigue^52^ ^,^ ^53^.

### Autoimmune and cholestatic liver diseases

Autoimmune hepatitis (AIH) is a chronic condition, more prevalent in young women, that requires immunosuppressive therapy. In these patients, HRQoL can be influenced by multiple factors, including disease activity, fatigue, depression, anxiety, side effects of immunosuppressive or corticosteroid treatment, concerns about disease progression, comorbidities, AST levels, incomplete biochemical remission, and, when present, cirrhosis^54^ ^,^ ^55^.

Chronic cholestatic diseases, such as primary biliary cholangitis (PBC) and primary sclerosing cholangitis (PSC), can also impair quality of life, mainly due to pruritus, fatigue, disease progression, anxiety, depression, ascites, and sexual and emotional dysfunction^56^ ^,^ ^57^ ^,^ ^58^. The use of ursodeoxycholic acid (UDCA) was associated with better quality-of-life scores^58^. A Canadian study found that HRQoL in PSC is lower than in the general population, as in other cholestatic diseases, and may be affected by several factors, including symptoms related to liver disease and inflammatory bowel disease, as well as decompensated cirrhosis^59^. Using SF-36 in 218 individuals with PSC, De Valle et al. found that the decrease in quality-of-life scores in these patients, compared to the general population, was mainly due to the domains of role functioning, general health, vitality, social functioning, role emotional, and mental health (all with *P*<0.05). The factors that negatively influenced HRQoL were advanced age, large duct disease, and systemic symptoms^60^. In addition to these findings, a longitudinal cohort study with 328 patients with PSC showed that IBD activity, advancing age, female gender, AIH overlap, pruritus, and end-stage liver disease were significantly associated with worse quality of life. Notably, during the post-transplant period, the individuals studied had HRQoL similar to that of the general population^61^.

### Other etiologies

Wilson’s disease (WD) is an autosomal recessive disorder of copper metabolism that can manifest with both hepatic and neuropsychiatric alterations, potentially impairing HRQoL. Studies have shown that psychiatric symptoms as the initial presentation, female sex, and treatment with zinc or trientine are associated with worse HRQoL^62^ ^,^ ^63^.

In hereditary hemochromatosis (HH), clinical and genetic factors can affect HRQoL, including fatigue, arthritis, cardiomyopathy, cirrhosis, and homozygosity for the p.Cys282Tyr mutation^64^ ^,^ ^65^.

### Comparison of HRQoL among different etiologies

Different liver diseases present distinct manifestations and complications that can variably impact quality of life. A Dutch study comparing HRQoL among patients with viral hepatitis, autoimmune hepatitis, cholestatic diseases, and hemochromatosis found that individuals with viral hepatitis experienced worse mental health parameters, while those with hemochromatosis suffered more from physical symptoms, particularly pain, compared to other etiologies^66^.

Sumskiene et al. evaluated 131 patients with cirrhosis and reported no differences in HRQoL among alcoholic, viral, primary biliary cholangitis (PBC), and primary sclerosing cholangitis (PSC) etiologies^67^. In contrast, Castellanos-Fernández et al., assessing 543 patients with chronic liver disease-including hepatitis B and C, steatotic disease, and autoimmune liver diseases-found significantly lower CLDQ scores in patients with steatotic disease compared to those with hepatitis B or autoimmune etiologies^68^. Gotardo et al., assessing patients listed for liver transplantation, observed that those with HCV cirrhosis had worse HRQoL than patients with alcohol-related cirrhosis, PBC, or autoimmune hepatitis (AIH)^69^.

Conversely, a Swedish study found no significant differences in SF-36 scores among cirrhotic patients with different etiologies, including hepatocellular, cholestatic, alcoholic, and HCV-related cirrhosis^70^. This heterogeneity may reflect cultural differences, variations in HRQoL instruments, and differences in sample characteristics.

### HRQoL and hepatocellular carcinoma

Hepatocellular carcinoma (HCC) is a common complication of liver cirrhosis and can be managed through various treatments, including surgical resection, chemoembolization, radioembolization, liver transplantation, and systemic therapies. Like other malignancies, HCC can further impair patients’ HRQoL across physical, psychological, and spiritual domains^71^. Factors associated with poorer HRQoL in HCC patients include advanced tumor stage, presence of cirrhosis, female sex, living alone, pain, fatigue, nausea and unemployment. Moreover, reduced HRQoL may serve as an independent prognostic factor for all-cause mortality in individuals with advanced HCC^72^ ^,^ ^73^. Interestingly, HRQoL data may also have prognostic value for mortality in HCC, as shown by Eilard et al.^74^. Treatment modality can influence HRQoL, although studies comparing techniques-including radiofrequency ablation (RFA), transarterial chemoembolization (TACE), transcatheter arterial embolization (TAE), liver resection, transarterial radioembolization (TARE), systemic therapy, and liver transplantation-have produced heterogeneous results, likely reflecting differences in patient populations and other variables^75^ ^-^ ^81^.

### HRQoL and liver transplant

Liver transplantation is the standard treatment for patients with advanced chronic liver disease, offering a one-year survival rate of up to 95% and substantially increasing life expectancy. It also significantly improves the quality of life. However, recipients require lifelong immunosuppression, which may cause side effects that negatively affect HRQoL^82^. Overall, transplantation enhances HRQoL by improving physical mobility, psychosocial functioning, social interactions, and independence in self-care, but this improvement may not be sustained in the long term^83^. Factors associated with a decline in post-transplant HRQoL include deterioration in mental and emotional health, episodes of acute cellular rejection, age over 60 years, osteoporosis, age above 50 at the time of transplantation, female sex, post-transplant complications, and underlying liver disease due to hepatitis C or alcohol^84^ ^,^ ^85^. In contrast, Duffy et al. evaluated patients who had undergone transplantation more than 20 years earlier and found that their overall HRQoL remained better than that of individuals living with chronic liver disease. Factors contributing to improved HRQoL in this long-term cohort included younger age, graft longevity, and adequate social support^86^. Casanovas et al., using the LDQOL questionnaire, found that certain domains-such as social functioning, vitality, sleep, social interaction, activities of daily living, and concerns about the future-did not improve after transplantation^87^. Additionally, sexual dysfunction, a key aspect of quality of life in patients with chronic liver disease, often persists in both men and women following transplantation^88^.

### HRQoL and nutritional aspects

Nutritional complications such as sarcopenia, frailty, and malnutrition frequently arise during the course of advanced chronic liver disease and may serve as prognostic indicators for both disease progression and patient quality of life^13^ ^,^ ^89^.

In a prospective observational study, García-Rodríguez et al. evaluated 110 individuals on the liver transplant waiting list and found that malnutrition risk could serve as a prognostic factor associated with transplant outcomes, anxiety, and quality of life^89^.

Frailty has been associated with poorer quality of life and is directly correlated with vitality, activities of daily living, emotional function, fatigue, physical functioning, and mental health^90^ ^,^ ^91^. Some studies have similarly demonstrated that sarcopenia independently contributes to reduced quality of life in patients with cirrhosis^92^ ^,^ ^93^. Several factors influence the quality of life in individuals with chronic liver disease and sarcopenia: albumin levels, emotional support, malnutrition, frailty, cramps, and disease severity, among others^94^.

### HRQoL and outcomes

Measuring quality of life may also help predict outcomes in patients with chronic liver disease. Macdonald et al. evaluated 405 individuals with cirrhosis and ascites and found that low scores on the physical component of the SF-36 were an independent predictor of 12-month mortality^95^. A Chinese cohort of 415 patients with chronic liver disease and 86 controls used the SF-36 questionnaire to assess HRQoL and found that mental and physical health scores behaved as predictors of survival^96^. In addition to predicting survival and mortality, HRQoL was independently associated with increased mortality/unplanned hospitalizations in patients with cirrhosis and could be an easy-to-use prognostic screen that patients could complete in the waiting room before their appointment^19^.

### HRQoL and psychosocial, emotional, and financial impacts

Patients with chronic liver disease experience negative effects on both emotional and financial well-being. Physical limitations can hinder work capacity, and combined with treatment-related expenses, these challenges can directly reduce quality of life due to economic losses. In the United States, the direct and indirect costs associated with cirrhosis reached approximately $2 billion in 2008^97^ and escalated to $9.5 billion by 2015^98^.

Additionally, factors such as stigmatization-often related to associations with alcohol use, intravenous drug use, obesity, or high-risk sexual behavior-marital status, anxiety, hopelessness, depression, occupation, and receipt of basic livelihood support can further influence HRQoL in this population^99^.

## CONCLUSION

Monitoring patients with chronic liver disease should extend beyond the implementation of therapeutic and prophylactic measures for disease-related complications. Physicians must recognize that, in addition to the clinical manifestations of disease progression, numerous other factors can negatively influence patients’ HRQoL, including the therapies and interventions themselves, social stigmatization, and psychological, social, economic, and spiritual challenges. A case-by-case assessment of these factors is essential, and management should adopt a multidisciplinary approach that addresses patients’ overall well-being while carefully weighing the risks and benefits of each treatment or intervention. Furthermore, consideration of the patient’s environment, the availability of social support, and the patient’s personal perspectives, concerns, and fears are crucial to ensure comprehensive care.

## Data Availability

Data in article: The research data are presented within the article itself (available in Tables 1, 2 and Figure 1).
